# Bee venom acupuncture alleviates trimellitic anhydride-induced atopic dermatitis-like skin lesions in mice

**DOI:** 10.1186/s12906-016-1019-y

**Published:** 2016-01-29

**Authors:** Bongjun Sur, Bombi Lee, Mijung Yeom, Ju-Hee Hong, Sunoh Kwon, Seung-Tae Kim, Hyang Sook Lee, Hi-Joon Park, Hyejung Lee, Dae-Hyun Hahm

**Affiliations:** 1 Acupuncture and Meridian Science Research Center, College of Korean Medicine, Kyung Hee University, Hoegi-ding, Dongdaemoon-gu, Seoul, 130-701 Republic of Korea; 2 Division of Meridian and Structural Medicine, School of Korean Medicine, Pusan National University, Yangsan, 628-870 Republic of Korea; 3 The Graduate School of Basic Science of Korean Medicine, College of Korean Medicine, Kyung Hee University, Seoul, 130-701 Republic of Korea

**Keywords:** Atopic dermatitis, Bee venom, Acupuncture, BL40, Trimellitic anhydride

## Abstract

**Background:**

Bee venom acupuncture (BVA), a novel type of acupuncture therapy in which purified bee venom is injected into the specific acupuncture point on the diseased part of the body, is used primarily for relieving pain and other musculoskeletal symptoms. In the present study, therapeutic potential of BVA to improve atopic dermatitis, a representative allergic dysfunction, was evaluated in the mouse model of trimellitic anhydride (TMA)-induced skin impairment.

**Methods:**

Mice were treated with 5 % TMA on the dorsal flank for sensitization and subsequently treated with 2 % TMA on the dorsum of both ears for an additional 12 days after a 3-day interval. From the 7^th^ day of 2 % TMA treatment, bilateral subcutaneous injection of BV (BV, 0.3 mg/kg) was performed daily at BL40 acupuncture points (located behind the knee) 1 h before 2 % TMA treatment for 5 days.

**Results:**

BVA treatment markedly inhibited the expression levels of both T helper cell type 1 (Th1) and Th2 cytokines in ear skin and lymph nodes of TMA-treated mice. Clinical features of AD-like symptoms such as ear skin symptom severity and thickness, inflammation, and lymph node weight were significantly alleviated by BV treatment. BV treatment also inhibited the proliferation and infiltration of T cells, the production of Th1 and Th2 cytokines, and the synthesis of interleukin (IL)-4 and immunoglobulin E (IgE)—typical allergic Th2 responses in blood. The inhibitory effect of BVA was more pronounced at BL40 acupoint than non-acupuncture point located at the base of the tail.

**Conclusions:**

These results indicate that BV injection at specific acupuncture points effectively alleviates AD-like skin lesions by inhibiting inflammatory and allergic responses in a TMA-induced contact hypersensitivity mouse model.

**Electronic supplementary material:**

The online version of this article (doi:10.1186/s12906-016-1019-y) contains supplementary material, which is available to authorized users.

## Background

In traditional Oriental medicine, bee venom acupuncture (BVA), a peculiar type of acupuncture, has been used to relieve pain and to treat various chronic inflammatory diseases in humans. It exerts not only pharmacological effects due to its various bioactive components, but also exerts an acupuncture effect by mechanical stimulation of acupuncture points [1].

BV seems to be much like a double-edged sword. In traditional Oriental medicine, BV therapy relieves pain and inflammation in various acute or chronic diseases [2]. On the other hand, it also induces a systemic or local allergic response, with fever, pain, and itching, sometimes leading to anaphylaxis [3]. According to clinical reports of BVA therapy, subcutaneous or intramuscular injection of BV often causes instant pain and inflammation around the injection site, as well as relieving pain and inflammation [4]. It has even been argued by Oriental physicians treating some musculoskeletal and immunological diseases using BV therapy that the greater the allergic response the better therapeutic effect is [5].

A growing number of studies provide compelling evidence for the anti-inflammatory effects of BVA in several animal pain models. For example, BVA can inhibit formalin-induced pain and carrageenan-induced inflammation in rat models [6, 7]. Recently, BV and its constituent, melittin, were reported to possess anti-inflammatory, antinociceptive, and anticancer effects, and also have a therapeutic effect against bacterial diarrhea in piglets [8–11]. BVA has also been used to relieve arthritic pain and edema in Korean traditional medicine [12]. Despite the many clinical and animal studies elucidating the medicinal effects of BVA in various pain and inflammatory diseases, there have been few studies of BVA as an alternative therapy for atopic diseases. Because BV sometimes induces systemic anaphylactic responses when injected in excessive amounts, it is reasonable to expect that diseases caused by immunological hypersensitivity, including atopic dermatitis, can be treated by honey bee venom, which is well known to be an inflammatory agent as well as an allergen. To the best of our knowledge, this is the first study to elucidate the medicinal effect of BV on atopic dermatitis-like skin disease in mice.

In this study, an atopic mouse model of allergen-induced contact hypersensitivity was used to assess BVA therapy for human atopic dermatitis. In contrast to other spontaneous (NC/Nga) transgenic and knockout mouse models, this model is simple to develop and its atopic symptoms are highly reproducible. Trimellitic anhydride (TMA), used in this study as an allergen, is a known respiratory sensitizer that induces T cell-dependent contact hypersensitivity in mice, eliciting eosinophil and T cell infiltration, T helper cell type 2 (Th2) cytokine production, and IgE release [13]. In the TMA-induced atopic model, mice are first sensitized on flank skin with TMA, and then T cell-dependent skin inflammation is induced by topical challenge with TMA on the dorsal surfaces of the ears 1 week after sensitization. The severity of atopic inflammation can easily be evaluated by observing ear appearance and measuring ear thickness. The effects of TMA-induced skin inflammation on the cutaneous cytokine profile, infiltration of immune cells, and serum IgE levels have been previously studied [14–16].

Although anti-inflammatory steroids are a conventional therapy for atopic dermatitis, there are however several concerns with this therapy, especially with long-term use. Mid- to high-potency steroids are contraindicated for use on the body and in intertriginous areas because of their side effects, including skin atrophy, hypopigmentation, striae, secondary infection, and acne [17]. Alternative treatments using BV are of particular interest, because BV seems to be effective without causing severe adverse effects like those that are often observed with steroid therapy.

For this purpose, we investigated the medicinal effect of BVA on atopic dermatitis using a TMA-induced contact hypersensitivity dermatitis mouse model. BVA injection was performed at the BL40 acupoint, which is known to cool down the body in Korean traditional medicine [18]. In addition to clinical observation, we also investigated the infiltration of immune cells in ear tissue by histological staining, serum IgE levels by enzyme-linked immunosorbent assay (ELISA), and T cell cytokine profiles in ears and lymph nodes using the Bio-Plex® suspension array system.

## Methods

### Animals

Male BALB/c mice weighing 28–30 g (10 weeks old) were purchased from Samtaco Co. (Osan, Korea). The mice were housed in a limited-access rodent facility with up to five mice per polycarbonate cage. They were housed in an air-conditioned animal room with a 12 h light/dark cycle (08:00–20:00 h light, 20:00–08:00 h dark) at 23 ± 2 °C and with 50 ± 10 % humidity. Mice were provided with a standard laboratory diet and water *ad libitum*. The animal experiments were conducted in accordance with the *Guide for the Care and Use of Laboratory Animals* (NIH Publication No. 80–23, revised in 1996), and were approved by the Kyung Hee University Institutional Animal Care and Use Committee. All animal experiments began at least 7 days after the animals arrived.

### Chemicals and drugs

All chemicals including TMA (98 %), bee venom, prednisolone, isopropyl myristate (98 %), dimethylsulfoxide, ethanol and corn oil were purchased from Sigma-Aldrich Co. (St. Louis, MO, USA). TMA was dissolved in a mixed solvent of acetone (Merck, Darmstadt, Germany) and isopropyl myristate (4:1, v/v) immediately before use. Bee venom was dissolved in saline, and prednisolone was dissolved in a mixed solvent of dimethylsulfoxide, ethanol and corn oil (5:3:92, v/v/v).

### Development of atopic dermatitis

A modified version of a protocol described by Schneider et al. was used to induce atopy-like skin dermatitis in mice [19]. Mice were first sensitized with 50 μL of 5 % TMA on the shaved dorsal flank skin on day 0. After an interval for 3 days, the animals received 10 μL of 2 % TMA once a day from days 3 to 14 on both sides of both ears. The mice were sacrificed under anesthesia with pentobarbital on the last day of the experiment. At autopsy, blood was collected from the retro-orbital plexus, and both ears and auricular lymph nodes were excised.

### Experimental groups

The mice were randomly divided into five experimental groups of ten animals each as follows: non-treated naive group (NOR, *n* = 10), vehicle-treated & TMA-treated atopic group (AD, *n* = 10), BV-treated at BL40 acupoint & TMA-treated atopic group (AD + BVA, *n* = 10), BV-treated at non-acupoint & TMA-treated atopic group (AD + BVNA, *n* = 10), and prednisolone-treated & TMA-treated atopic group (AD + PRE, *n* = 10). Prednisolone (30 mg/kg, p.o.) was used as a positive control.

### BVA and drug treatments

To determine the optimum conditions for BV injection, changes in skin temperature at the injection site were analyzed under conditions of subcutaneous, intradermal, and intramuscular injection of BV. BV (0.3 mg BV/kg body weight) was injected into the mid-back after hair removal, and the temperature at the injection site was measured using an infrared thermometer (HuBDIC Thermofinder (FS-300), Beauty Korea World Co., Ltd., Seoul, Korea). Each type of BV injection was performed in at least three mice, and temperature measurement was repeated three times per injection site per time point.

**Fig. 1:**
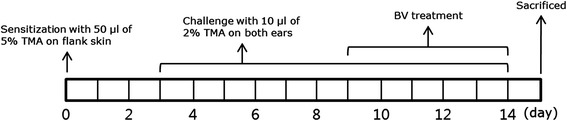
Experimental schedules for developing TMA-induced atopic dermatitis and treating with BV in mice. TMA, trimellitic anhydride; BV, bee venom

Daily treatment with BV and prednisolone was performed 1 h before TMA challenge on days 9 to 14 (a total of six times) (Fig. 1). For BVA treatment, the mice were gently immobilized with hands, and 20 μL BV solution (0.3 mg/kg) was injected bilaterally at a depth of 1 mm at the BL40 acupoints, located in the center of the popliteal crease between the tendons of the biceps femoris and semitendinosus muscles on both legs, using a 1 mL insulin syringe (needle gauge 26; BD Biosciences Co., CA, USA). A non-acupuncture point at the base of the tail was used as a control. The needles were removed from the acupoints immediately after BV injection. As a positive control, prednisolone, a glucocorticoid prodrug, was administered orally to mice at a dose of 30 mg/kg body weight.

### Scoring of ear skin manifestations

The ear skin of mice in each experimental group was photographed using a digital camera (Canon 20D; Canon Inc., Tokyo, Japan) to analyze changes in atopic symptoms and clinical appearance of the ear skin and tissues. The severity of atopic dermatitis was macroscopically assessed, and scored in a blinded fashion every 2 days starting from the day after the first 5 % TMA challenge, according to the protocol of Shin HK et al. [20]. Atopic symptoms of ear skins were evaluated by scoring scaling and dryness, hemorrhage and excoriation, and edema and redness, and by then calculating the sum of the individual symptom scores for both ears, graded as 0 (no symptoms), 1 (mild), 2 (moderate), or 3 (severe). The total score for each animal ranges from 0 to 9.

### Measurements of ear thickness and auricular lymph node weight

Ear thickness and auricular lymph node weights of all 10 mice in each experimental group was measured. Ear thickness was measured with a dial thickness gauge (Ozaki Seisakusho Co., Tokyo, Japan), and the weight of the auricular lymph node was measured with a digital balance (Mettler-Toledo Inc., Columbus, OH, USA).

### ELISA of immune mediators

After scoring of ear skin manifestation, blood samples were rapidly collected from all 10 mice in each experimental group under anesthesia on the day of killing via the retro-orbital plexus using a capillary pipette. Serum was obtained from the blood samples by centrifugation at 6500 rpm for 20 min, and was stored at −70 °C until use. Serum IL-4 and IgE levels were measured using an ELISA kit (R&D Systems Inc., MN, USA and Bethyl Laboratories Inc., TX, USA, respectively). After coating the inner surfaces of microplate wells with each antibody at 4 °C overnight, serum samples (50 μL, 3-fold dilution) were dispensed into the wells and incubated for 1 h. After washing the plate twice, 100 μL avidin-horseradish peroxidase (HRP)-conjugated antibody was added. After another wash, tetramethylbenzidine (TMB) solution was added, and the plate was incubated for 30 min in the dark. After stopping the reaction with 50 μL stop solution, the absorbance at 450 nm was measured using an ELISA reader (Multi-Read 400; Authos Co., Austria).

### RT-PCR analysis of immune mediators

For this, 5 mice from each group (*n* = 10) were deeply anesthetized with sodium pentobarbital (50 mg/kg, i.p.), and their ear tissues and auricular lymph nodes were quickly collected. Total RNA was isolated from each tissue sample of ear or lymph node. After excision, tissue samples were quickly stored at −80 °C until use. Total RNA was isolated using TRIzol® reagent (Gibco BRL Co., MD, USA). Complementary DNA (cDNA) was synthesized from total RNA with reverse transcriptase (Takara Co., Shiga, Japan). The expression levels of mRNAs were determined by PCR analysis using cDNA as the template. PCR was performed using a PTC-100 programmable thermal controller (MJ Research Co., MA, USA). Primers were designed based on published mRNA sequences using Primer3 primer selection software (Whitehead institute for Biomedical Research, Cambridge, MA: http://biotools.umassmed.edu/bioapps/primer3_www.cgi). Glyceraldehyde 3-phosphate dehydrogenase (GAPDH) was used as a housekeeping gene. PCR products were separated on 1.2 % agarose gels and stained with ethidium bromide. Subsequently, band densities were analyzed using an image analysis system (i-Max™; Core Bio System Co., Ltd., Seoul, Korea). Relative gene expression was determined by calculating the relative band intensity for each gene compared to GAPDH. Table 1 provides the primer sequences and annealing conditions for PCR.

**Table 1:** Nucleotide sequences of primers and operating condition for PCR analysis

Gene		Nucleotide sequence	Operating condition
GAPDH	sense	5′-AACTTTGGCATTGTGGAAGG-3′	94 °C, 30s 58 °C, 30s 72 °C, 30s, 30 cycles
	antisense	5′-ACACATTGGGGGTAGGAACA-3′	
IL-1β	sense	5′-GGCTGTGGAGAAGCTGTGGC-3′	
	antisense	5′-GGGTGGGTGTGCCGTCTTTC-3′	
TNF-α	sense	5′-GCAGAAGAGGCACTCCCCCA-3′	
	antisense	5′-GATCCATGCCGTTGGCCAGG-3′	
IL-4	sense	5′-TCAACCCCCAGCTAGTTGTC-3′	
	antisense	5′-TGTTCTTCGTTGCTGTGAGG-3′	

*T* thymine, *A* adenine, *C* cytosine, *G* guanine, *GAPDH* glyceraldehyde-3-phosphate dehydrogenase, *IL* interleukin, *TNF* tumor necrosis factor

### Histology and immunohistochemistry

Five mice from each group (*n* = 10) were deeply anesthetized with sodium pentobarbital (50 mg/kg, i.p.), and their ear tissues were quickly collected. Each ear tissue was embedded in paraffin, and cut into 6 μm-thick sections using a rotatory microtome (Finesse 325; Thermo Shandon Co., UK). The sections were deparaffinized before staining. To demonstrate morphologic changes and eosinophil infiltration, sections were stained with hematoxylin (Merck, Darmstadt, Germany) and 1 % eosin (Sigma-Aldrich Co.) [21]. Staining with toluidine blue (Merck) was performed for mast cell detection. For immunohistochemistry, the other half of each ear was embedded in paraffin and cut into 6 μm-thick sections. The sections were deparaffinized before immunohistochemistry. Slides were incubated overnight at 4 °C in a primary antibody solution containing anti-mouse cluster of differentiation (CD)4 and anti-mouse CD8 rabbit antibodies (1:200 dilution; Novus Biologicals Co., Littleton, USA), after which they were incubated with anti-rabbit secondary antibody (1:500 dilution; Vector Laboratories Inc., CA, USA). Next, the slides were treated with a Vectastain™ Elite ABC kit (Vector Laboratories Inc.). Immunopositive spots on the slides were developed using diaminobenzidine (DAB) as a colorimetric substrate. A cover slip was then placed over the tissue. All slides were examined at 100× magnification using a microscope equipped with a digital camera (BX51; Olympus Co., Tokyo, Japan) and DP2-BSW analysis software (Olympus Co).

### Bio-Plex analysis of Th1 and Th2 cytokines in auricular lymph node tissue

Five mice from each group (*n* = 10) were deeply anesthetized with sodium pentobarbital (50 mg/kg, i.p.), and their auricular lymph nodes were quickly collected. Cytokine assay of each auricular lymph node was performed using the Bio-Plex Mouse Cytokine 8-Plex Panel (one 96-well plate) (Bio-Rad Laboratories, Inc., CA, USA) according to the manufacturer’s instructions. This is a multiplex bead-based assay (xMAP Technology) involving diverse matrices that are designed to simultaneously quantitate many cytokines in a small amount of tissue. The assay was performed as follows. The wells of a 96-well filter plate were pre-wetted with 100 μL Bio-Plex assay buffer. Multiplex bead working solution was vortexed for 15–20 s at medium speed, and 50 μL solution was added to each well. The buffer was removed by vacuum filtration and 100 μL fresh Bio-Plex wash buffer was added to each well. The buffer was again removed by vacuum filtration. This step was repeated once again and 50 μL diluted standard or sample was added to each well. The plate was covered with aluminum foil and shaken at 1100 rpm for 30 s, and then shaken at 300 rpm for 90 min at room temperature. Next, the plate was washed three times with 100 μL Bio-Plex wash buffer. After vacuum filtration, vortexed working solution of Bio-Plex Detection Antibody (25 μL) was gently added to each well, and the plate was shaken as described above and washed three times with Bio-Plex washing buffer. Vigorously vortexed 1× streptavidin-peroxidase solution (50 μL) was added to each well, and the plate was shaken at 1100 rpm for 30 s, and subsequently at 300 rpm for 10 min. After three washes, the beads in each well were resuspended with 125 μL Bio-Plex assay buffer, and the plate was shaken at 1100 rpm for 30 s. Beads were read using Bio-Plex Manager® software.

### Statistical analysis

All measurements were performed by an independent investigator blinded to the experimental conditions. Results in figures are expressed as mean ± standard error of means (SEM). Experimental data were analyzed by one-way ANOVA using SPSS version 13.0 (IBM, Chicago, USA). Statistical differences among groups were further analyzed using Tukey’s post hoc test. All p values less than 0.05 were considered statistically significant.

## Results

### Clinical manifestation of TMA-treated atopy-like ear skin lesions

**Fig. 2:**
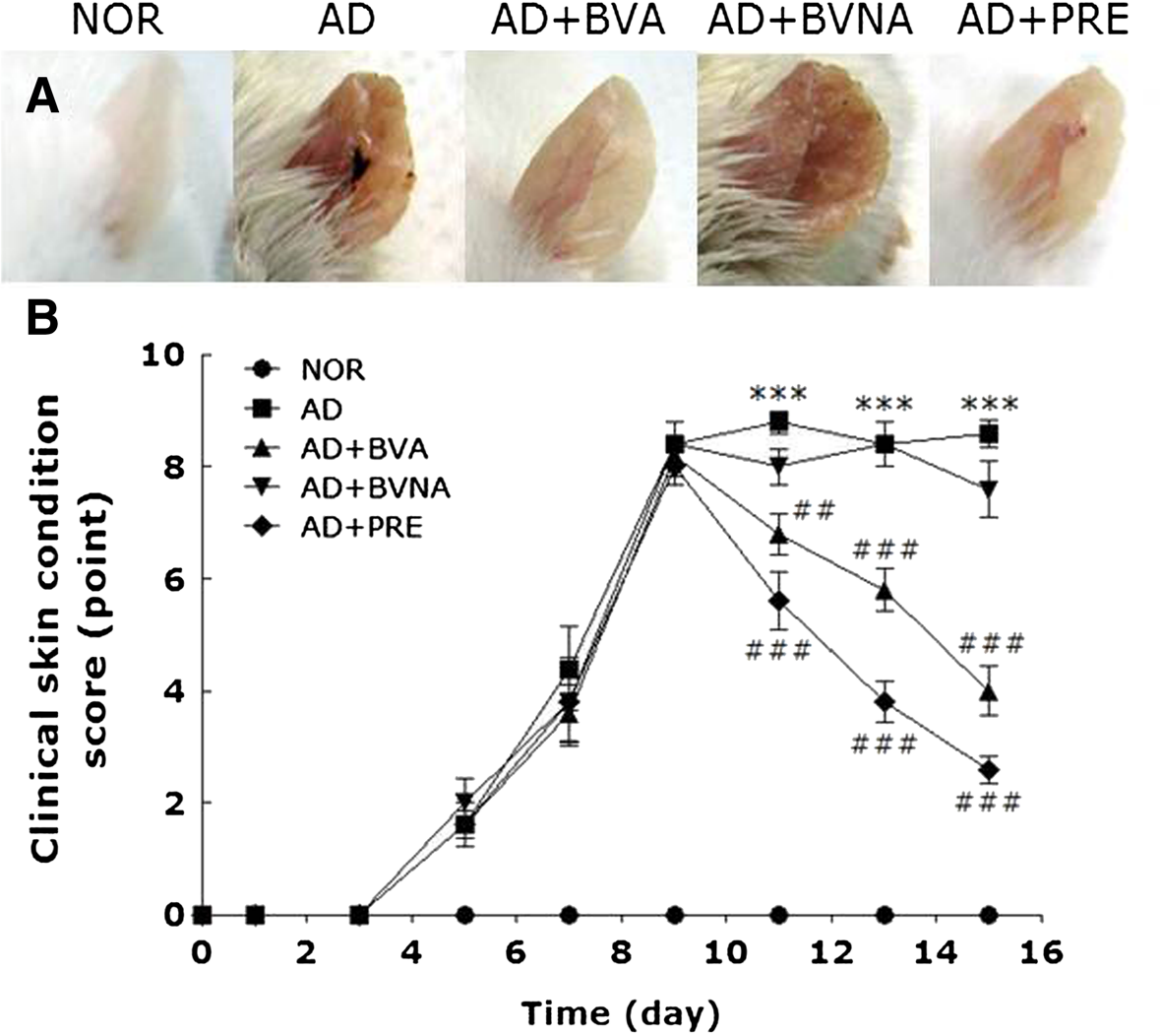
Representative images (**a**) of mouse ears and scoring graph (**b**) in the NOR, AD, AD + BVA, AD + BVNA and AD + PRE groups. The graph indicates time-course severities of atopic dermatitis by scoring each image between 0 and 9 points depending on the following skin symptoms: scaling and dryness, hemorrhage and excoriation, and edema and redness. The arrow indicates the initiation of BV or prednisolon treatment. ^***^ *p* < 0.001 vs. the NOR group; ^##^ *p* < 0.01, ^###^ *p* < 0.001 vs. the AD group

Repeated application of TMA to mouse ear skins induced atopy-like skin lesions with typical atopic symptoms such as erythema, excoriation, erosion, scaling, and dryness (Fig. 2a). The severity of atopic disease was evaluated by individually scoring the symptoms (skin dryness, hemorrhage and excoriation, and edema and redness). The atopic dermatitis-like skin symptoms began to be observed on day 3 during the 5 % TMA challenge period, and showed maximum exacerbation on day 9 during the 5 % TMA challenge period, as shown in Fig. 2b. BV treatment was started on day 9, when atopy-like symptoms reached their peak. Daily BV treatment at the BL40 acupoint markedly alleviated the symptoms without apparent initial signs of acute inflammation or hypersensitivity due to BV injection. There were also no inflammation responses in the skin tissues around the BL40 acupoint after BV injection (data not shown). The therapeutic efficacy of BVA at BL40 was similar to that of prednisolone, a steroid drug, which was used as a positive control in the present study. Acupuncture stimulation at a non-acupuncture point (AD+ BVNA) showed no therapeutic effect on atopic dermatitis-like symptoms in mice.

### Changes in ear thickness and auricular lymph node weight

**Fig. 3:**
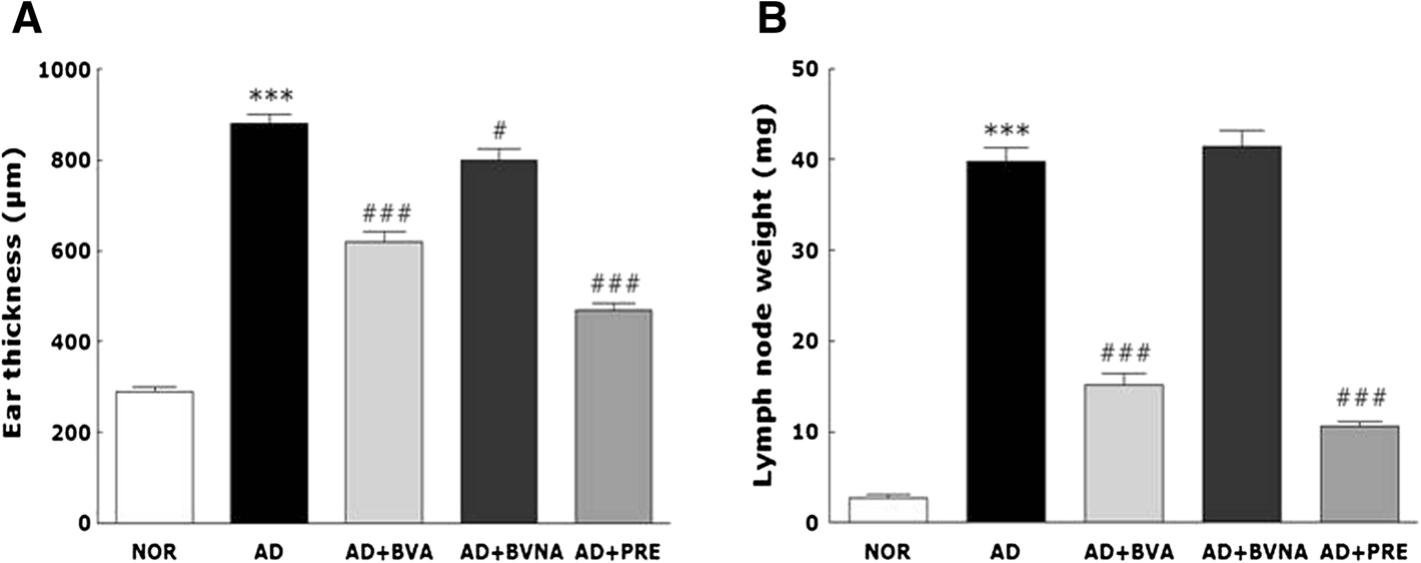
Ear thickness (**a**) and lymph node weights (**b**) of the mice in the NOR, AD, AD + BVA, AD + BVNA and AD + PRE groups. ^***^ *p* < 0.001 vs. the NOR group; ^#^ *p* < 0.05, ^###^ *p* < 0.001 vs. the AD group

In addition to morphological changes in inflamed ear skin, repeated application of TMA to the ear skin also induced a significant increase in ear thickness in mice (Fig. 3a). Mouse ear thickness increased about 3-fold by TMA treatment, and BV injection at acupoint BL40 significantly suppressed the increase in ear thickness on day 14 (*p* < 0.001). The medicinal efficacy of BVA treatment on reducing ear thickness in the AD + BVA group was similar to that of prednisolone in the AD + PRE group. Although BVA stimulation at a non-acupuncture point also reduced ear thickness, the effect was negligible, as compared to that of acupuncture stimulation at BL40. As shown in Fig. 3b, repeated treatments of TMA in the AD group induced a significant increase in auricular lymph node weight.

Auricular lymph node weight on day 14 after TMA treatment increased about 13-fold. Whereas BV injection at a non-acupuncture point on the tail had no effect on the increase in auricular lymph node weight, the same treatment at acupoint BL40 significantly reduced auricular lymph node weights (*p* < 0.001). Moreover, the decrease in auricular lymph node weight in the AD + BVA group was similar to that in the AD + PRE group, a positive control group.

### Changes of serum IL-4 and IgE levels

**Fig. 4:**
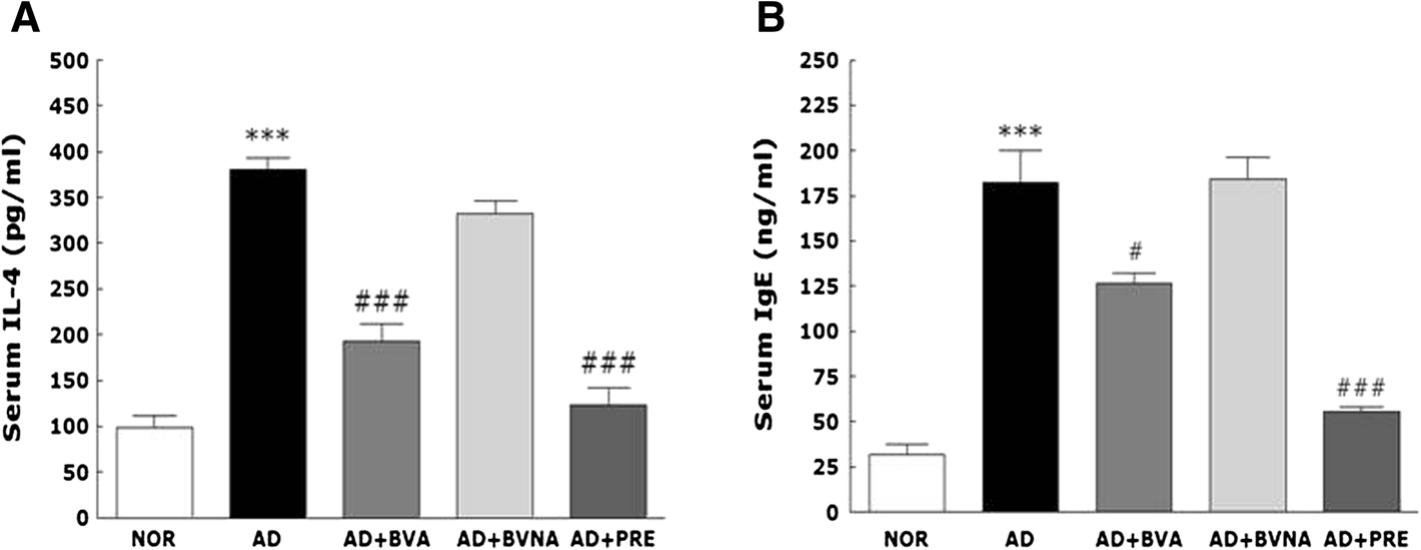
Serum levels of IL-4 (**a**) and IgE (**b**) in the NOR, AD, AD + BVA, AD + BVNA and AD + PRE groups using an ELISA. IL, interleukin; IgE, immunoglobulin E; ELISA, enzyme-linked immunosorbent assay. ^***^ *p* < 0.001 vs. the NOR group; ^#^ *p* < 0.05, ^###^ *p* < 0.001 vs. the AD group

Serum levels of IgE and IL-4, which are increased in most patients with atopic dermatitis, were determined in the TMA-induced mouse model as a parameter for humoral (Th2) responses [22, 23]. In the present study, as shown in Fig. 4, serum levels of IL-4 and IgE were elevated about 3.7-fold and 6.5-fold, respectively, in the AD group on day 14 after TMA treatment. The secretion profiles of these factors are very similar to the profile for eosinophil activation, which plays a crucial role in aggravating atopic dermatitis symptoms [24]. BV injection at acupoint BL40 significantly suppressed the elevation of IL-4 and IgE levels (*p* < 0.001) on day 14. Notably, the suppressive effect of BV treatment at acupoint BL40 on serum IL-4 level in the AD + BVA group was comparable to that in the AD + PRE group. BV injection at a non-acupuncture point on the tail did not reduce serum IgE and IL-4 levels.

### Histological changes of ear skin tissues

**Fig. 5:**
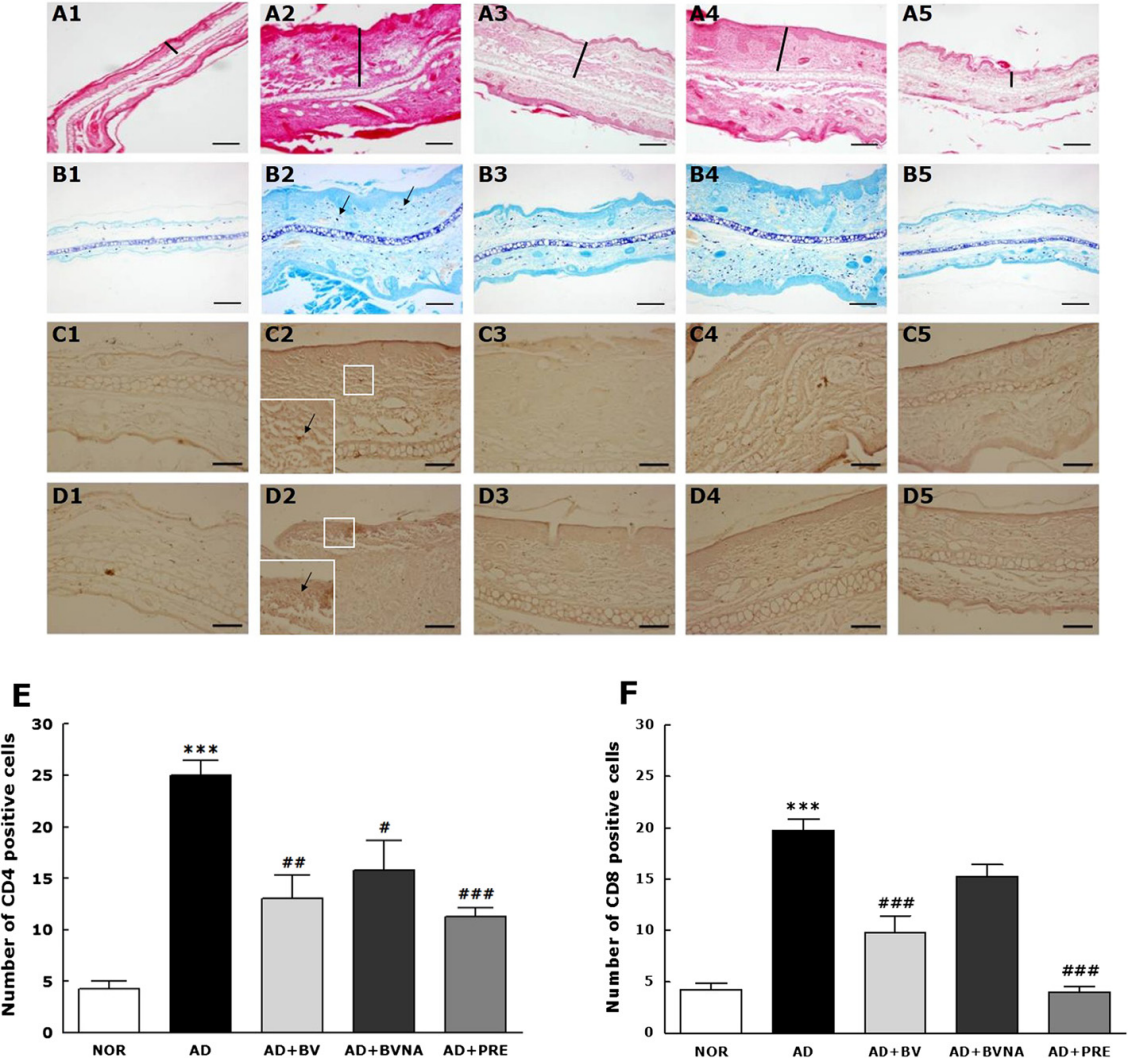
Histological images and graphs indicating relative percentage of CD4- and CD8-immunopositive cells of the ear sections. Ear sections in each group were stained with hematoxylin and eosin (**a**1-**a**5), toluidine blue (**b**1-**b**5), anti-mouse CD4 IgG (**c**1-**c**5) and anti-mouse CD8 IgG (**d**1-**d**5). Black scale bar indicates 100 μm (100× magnification). Black thick lines in the images of H-E staining indicate the thickness of ear skins. Small white squares (200×) in the centers of immunohistological staining images (**c**2 & **d**2 of AD group) are magnified in the lower left corners to observe CD4- and CD8- positive cells infiltrated into the skin tissues. The representative CD4- and CD8-immunopositive cells were indicated by black arrows in indicated in small white squares in C2 and D2, respectively. The numbers of CD4- and CD8-immunopositive cells in the fixed area of the images are depicted in the bar graphs **e** and **f**, respectively, below the histological images. IgG, immunoglobulin G; CD, cluster of differentiation. ^***^ *p* < 0.001 vs. the NOR group; ^#^ *p* < 0.05, ^##^ *p* < 0.005 and ^###^ *p* < 0.001 vs. the AD group

Morphological changes in inflamed skin layers and cutaneous infiltration of immune cells were characterized by histochemical techniques (Fig. 5). Inflammatory responses such as thickening of ear skin including dermis and epidermis, edema and epidermal hyperplasia, which leads to massive infiltration of various immune cells from the blood vessels into the dermis layers, were observed in coronal sections of ear skin tissues stained with hematoxylin and eosin in the AD group (Fig. 5a1-a5). BV treatment at acupoint BL40 significantly inhibited these inflammatory changes in both the epidermis and dermis, although its suppressive efficacy was lower than that of prednisolone. BVA therapy at a non-acupuncture point had no significant effect on histochemical changes in inflammation.

Of the pathogenic features of blood in atopic dermatitis patients, such as IgE, eosinophils, and mast cells, the role of mast cells is demonstrated by increases in cell numbers and mast cell activation in atopic dermatitis lesions [25]. In the present study, mast cells in the dermis in mice with TMA-induced atopy-like dermatitis were analyzed by toluidine blue staining (Fig. 5b1-b5). Topical application of TMA markedly increased mast cell infiltration in the epidermis and dermis of ear skin in the AD group. BV treatment at acupoint BL40 significantly alleviated the TMA-induced infiltration of mast cells. Its therapeutic efficacy was similar to that of prednisolone. BV stimulation at a non-acupuncture point had no such effect.

To investigate cutaneous infiltration of allergen-specific T cells, CD4^+^, and CD8^+^ T cells were analyzed in ear skin tissues using an immunohistochemical technique (Fig. 5c1-c5 and d1-d5). In the present study, CD4^+^ T cell infiltration was predominantly observed in the dermis and CD8^+^ T cells were mainly localized in the epidermis in the AD group, as shown in Fig. 5c2 and d2. BV injection at acupoint BL40 significantly inhibited the infiltration of CD4^+^ and CD8^+^ T cells caused by repeated application of TMA (Fig. 5e and f). Its therapeutic efficacy was similar to that of prednisolone. BV stimulation at a non-acupuncture point had no effect of suppressing the infiltration of CD8^+^ T cells even though there was little effect of non-acupoint stimulation in case of CD4^+^ T cells.

### RT-PCR analysis of cytokine mRNA expression in ear skin and auricular lymph nodes

**Fig. 6:**
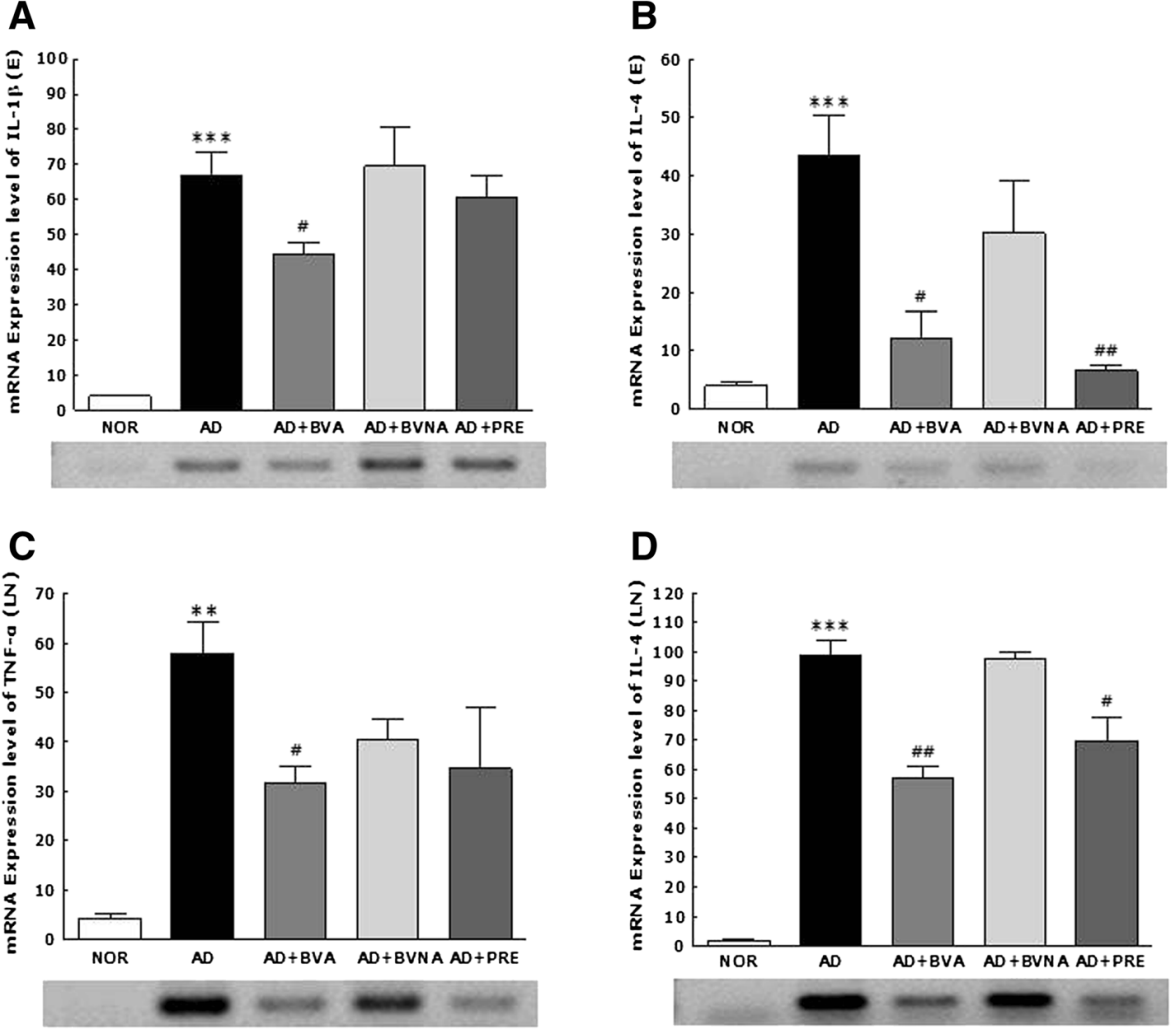
The mRNA expression levels of IL-1β (**a**) and IL-4 (**b**) in ear tissue (E), and TNF-α (**c**), and IL-4 (**d**) in auricular lymph node (LN) in mice. IL, interleukin; TNF, tissue necrosis factor. ^**^ *p* < 0.01, ^***^ *p* < 0.001 vs. the NOR group; ^#^ *p* < 0.05, ^##^ *p* < 0.01, ^###^ *p* < 0.001 vs. the AD group

To investigate Th1 and Th2 cytokine gene expression, mRNA levels of cytokines such as tumor necrosis factor (TNF)-α, IL-1β, and IL-4 were determined in ear and auricular lymph node tissue homogenates using RT-PCR (Fig. 6). TNF-α and IL-1β were selected as Th1 inflammatory cytokines for the auricular lymph node and ear, respectively, and IL-4 was selected as a representative Th2 cytokine for both tissues. In ear tissues, mRNA expression of IL-1β and IL-4 showed similar patterns: repeated application with TMA induced significant increases in the expression of IL-1β and IL-4 mRNAs, and BV treatment at acupoint BL40 significantly suppressed the TMA-induced mRNA expression of both cytokines. The suppressive effect of BV treatment at acupoint BL40 was similar to that of prednisolone. In all cases, BV stimulation at a non-acupuncture point showed weaker suppressive effects even though suppression levels were insignificant than that in the BL40 acupoint stimulation except IL-1β.

In auricular lymph node tissues, topical application of TMA induced significant increases in the expression of TNF-α and IL-4 mRNAs. BV treatment at acupoint BL40 significantly suppressed TMA-induced mRNA expression of both cytokines in the lymph node. In the case of TNF-α, application of BVA to a non-acupuncture point had a non-significant suppressive effect on mRNA expression of the cytokines.

### Bio-Plex analysis of Th1 and Th2 cytokine production in the auricular lymph node

**Fig. 7:**
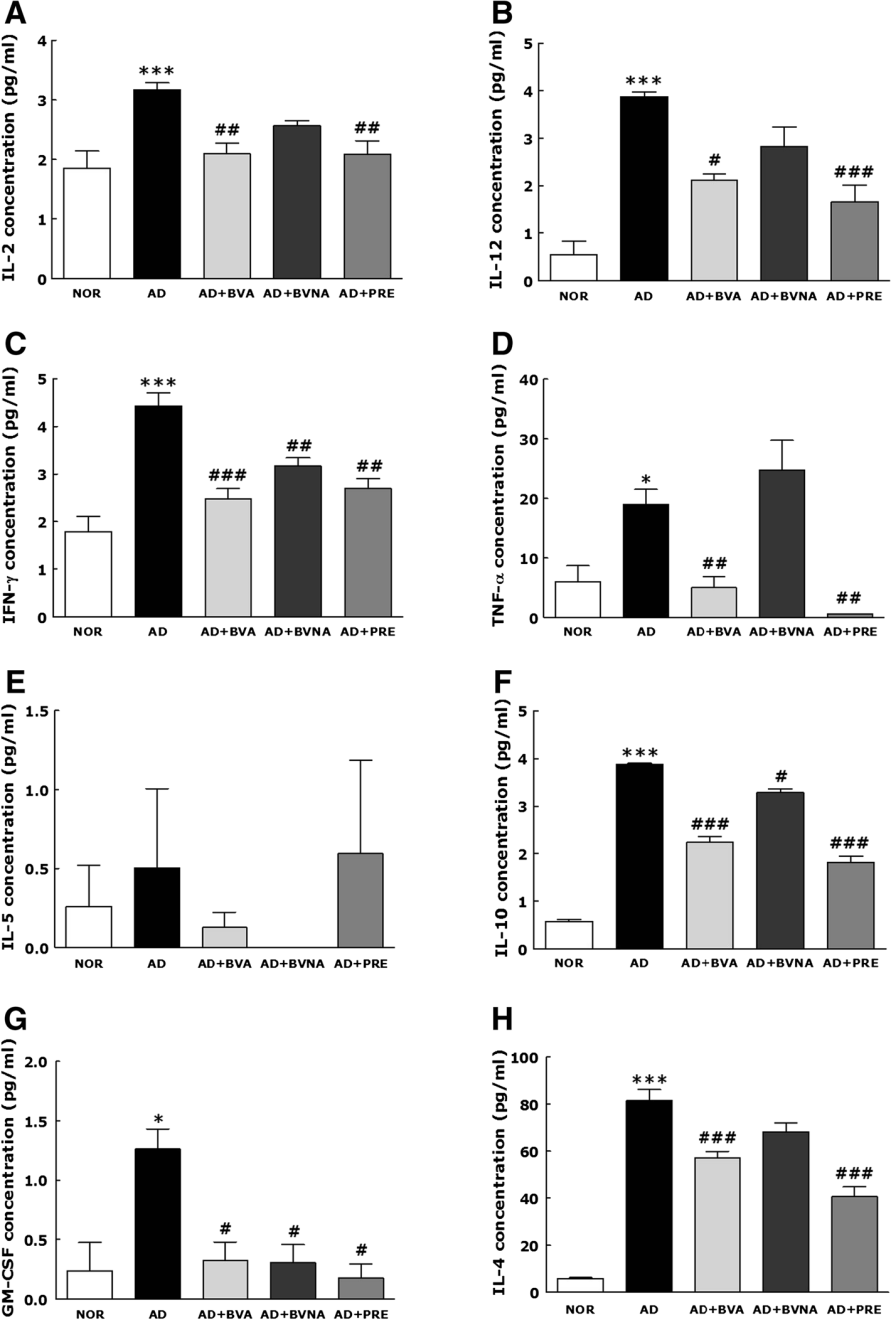
Analysis of the levels of cytokines such as IL-2(**a**), IL-12(**b**), IFN-γ(**c**), IL-5(**d**), IL-10(**e**), GM-CSF(**f**), TNF-α(**g**) and IL-4(**h**). The analysis was performed using the Bio-Plex® suspension array system, in the auricular lymph node in mice. GM-CSF, granulocyte macrophage colony-stimulating factor; IFN, interferon; IL, interleukin; TNF, tissue necrosis factor. ^*^ *p* < 0.05, ^***^ *p* < 0.001 vs. the NOR group; ^#^ *p* < 0.05, ^##^ *p* < 0.01, ^###^ *p* < 0.001 vs. the AD group

When allergens including TMA activate naïve anti-allergen T cells in mice, lymph node IL-4 concentrations are abruptly increased, and subsequently induce naïve T cells to differentiate into Th2 effector cells. These Th2 cells then secrete Th2 cytokines to promote isotype switching to IgE in activated B cells, and to influence other immune cells responding to the allergen [26, 27]. In the present study, the secretion profiles of cutaneous cytokines in auricular lymph node tissues were measured at the protein level using the Bio-Plex suspension array system (Fig. 7). IL-2 (a), IL-12 (b), interferon (IFN)-γ (c) and TNF-α (d) were quantified as Th1 inflammatory cytokines, and IL-5 (e), IL-10 (f), granulocyte-macrophage colony-stimulating factor (GM-CSF) (g) and IL-4 (h) as Th2 cytokines in auricular lymph node tissues. Repeated challenge with TMA caused significant increases in the secretion of all cytokines, regardless of Th1/Th2 phenotype. Among the cytokines examined, IL-5 showed the weakest induction after repeated topical application of TMA, and IL-4 showed the strongest. BV treatment at acupoint BL40 significantly suppressed the TMA-induced increases in the secretion of these cytokines. BV treatment at a non-acupuncture point had a little significant suppressive effect on protein expression of the cytokines such as IFN-γ, IL-10 and GM-CSF while there were also non-significant effects of suppression in cases of IL-2, IL-4, IL-5 and IL-12 expression. In the AD + BVA group, the protein expression levels of the Th1 cytokines IL-2, IL-12, IFN-γ, and TNF-α decreased to 2.1 ± 0.17 (*p* < 0.01, AD group vs. AD + BVA group), 2.12 ± 0.13 (*p* < 0.05, AD group vs. AD + BVA group), 2.48 ± 0.21 (*p* < 0.001, AD group vs. AD + BVA group), and 5.04 ± 1.76 (*p* < 0.01, AD group vs. AD + BVA group), respectively, from 3.17 ± 0.12, 3.88 ± 0.1, 4.43 ± 0.27, and 19.0 ± 2.5 in the AD group. In the AD + BVA group, the protein expression levels of the Th2 cytokines IL-5, IL-10, GM-CSF, and IL-4 decreased to 0.13 ± 0.1, 2.23 ± 0.12 (*p* < 0.001, AD group vs. AD + BVA group), 0.33 ± 0.15 (*p* < 0.05, AD group vs. AD + BVA group), and 57.23 ± 2.7 (*p* < 0.001, AD group vs. AD + BVA group), respectively, from 0.5 ± 0.5, 3.87 ± 0.03, 1.26 ± 0.17, and 81.64 ± 4.72 in the AD group. The therapeutic efficacy of BVA treatment at suppressing the secretion of TNF-α and IL-2 (Th1 cytokines), and IL-4, IL-10, and GM-CSF (Th2 cytokines), was similar to that of prednisolone.

## Discussion

There have been few objective ways to determine the optimum dose of BV in clinics and animal studies. According to unpublished reports from Oriental clinics, the best outcome with BVA can be achieved with the maximum dose of BV, with substantial heat and pain around the injection site. Because the injection of excessive amounts of BV sometimes induces anaphylactic symptoms, physicians using BVA gradually increase its dose according to the patient’s verbal response to treatment. One possible way to objectively optimize the injection method or daily dose is to measure the skin temperature around the BV injection site. We measured the temperature at the injection site after intradermal, subcutaneous, and intramuscular injection of BV into the backs of mice, and eventually adopted subcutaneous injection for BVA in this study (Additional file 1: Figure S1).

In the present study, BV therapy was used to treat atopic dermatitis, a typical allergic disease in which the intrinsic immune system is over-activated in response to various environmental factors. To investigate anti-inflammatory activities of BVA, we produced chronic TMA-induced T cell-dependent skin inflammation in mouse ears using the protocol of Schneider et al. [19]. In this model, the repeated challenge of ear skin with 2 % TMA for 12 days after one-shot sensitization with 5 % TMA on day 0 caused atopic dermatitis-like skin symptoms, such as increased skin thickness, change in skin morphology, and infiltration of immune cells [28]. It is widely accepted that eosinophils, mast cells, and CD4^+^ T cells mainly infiltrated the dermis and CD8^+^ T cells mainly infiltrated the epidermis [29]. And our results were also coincident with the previous findings. From histological findings that the majority of skin-infiltrating T cells in active atopic dermatitis lesions in humans are CD4^+^ T cells, CD4^+^ T cells are considered pivotal to the development of eczema and skin eosinophilic inflammation, because they produce a mixed pattern of Th1 and Th2 cytokines in the pathological development of atopic dermatitis [26]. CD8^+^ T cells are also dominant effector cells. They are responsible for allergen-induced skin inflammation, and thus their infiltration is required for the development of atopic dermatitis-like lesions and the initiation of mixed Th1/Th2 skin inflammation in rodent models.

Local lymph node weight is also increased by topical application of allergens including TMA or haptens such as dinitrofluorobenzene, 2,4-dinitrochlorobenzene, and trinitrochlorobenzene [30]. Hence, local lymph node weight was recently validated as a main index of allergic and immune responses in murine models. In the present study, lymph node weight also significantly increased after repeated challenge of ear skin with TMA (Fig. 3b).

Repeated topical application of allergens or haptens causes significant increases in blood IgE and IL-4 levels in a rodent allergy model [23, 31]. Topical exposure of haptens including dinitrofluorobenzene, 2,4-dinitrochlorobenzene (DNCB), and trinitrochlorobenzene (TNCB) also developed severe contact hypersensitivity in the mice. However, unlike allergens including TMA, the significant increases in eosinophil and T-cell infiltration, Th2 cytokine production and IgE levels were not observed in the serum of these hapten-induced hypersensitivity animal models, although a minor increase was reported previously [32].

In the present study, TMA treatment directly triggered a T cell-mediated contact hypersensitivity reaction and also caused distinct changes in the innate and adaptive immune systems, primarily indicated by increases in the secretion of Th1 and inflammatory cytokines, and the production of Th2 cytokines and IgE, respectively.

Serological and tissue investigations also showed an increased serum IgE level and Th2 cytokine dominance in the serum, ear skin, and auricular lymph node, despite the simultaneous dominance of Th1 cytokines such as TNF-α, IL-1β, IL-2, IL-12, and IFN-γ in ear or lymph node tissues. In ear and auricular lymph node tissues in our atopic animal model, Th1 cytokines were strongly secreted, although Th2 cytokines dominated. This might be due to a longer period of 2 % TMA challenge on both ears (3 days longer than in Schneider’s protocol) to elicit an intensified immune response.

As an alternative therapy, BV was injected to stimulate a specific skin point behind the knee. The stimulation point is located far away from the ears where allergic inflammation is induced by repeated exposure to TMA, and a specific component of BV can hardly be responsible for alleviation of skin symptoms. Thus, our results demonstrate that stimulation of the specific acupoint by BV significantly inhibited TMA-induced skin inflammation in the ears, probably due to modulation of the systemic immune system. Although the detailed mechanism has not yet been elucidated, the improvement in symptoms must be attributed to certain systemic changes being able to affect every part of the body as well as being triggered by local BV stimulation.

On the other side, as reported previously, BV contains several biologically active peptides, including melittin, apamin, and adolapin [33, 34]. Many studies indicate that BVA can modulate a systemic immune response by inhibiting inflammatory mediators, similar to non-steroidal anti-inflammatory drugs (NSAIDs) [35]. Other studies reported that BV has in vitro anti-inflammatory activity which is ascribed to the transcriptional downregulation of NF-κB and MAPKs in target tissues [36]. One can speculate that Th1 cytokine-based inflammatory symptoms in atopic dermatitis were alleviated by BV components and that this pharmacological anti-inflammatory effect was exerted via modulation of NF-κB and MAPK signal transduction pathways.

Since atopic dermatitis is primarily characterized by skin symptoms such as erythema, edema, excoriation, and scaling, we first investigated the therapeutic effects of BVA on ear edema and thickness in our animal model [37]. At the maximum severity of AD-like symptoms, we started BVA treatment at a dose of 0.3 mg/kg at the BL40 acupoints on both legs and found significant reductions in ear edema and thickness 2 days after starting BVA treatment. We thereafter confirmed that BVA treatment was very effective at inhibiting activation of resident mast cells and infiltration of immune cells by histological analysis. Because mast cells play a key role in mediating allergic inflammation in asthma, atopic dermatitis, and sinusitis, we also focused on changes in mast cells in the dermis of inflamed skin. BV treatment significantly decreased mast cell infiltration in ear tissues [38]. Thus, it is likely that BV regulates the infiltration of mast cells and inhibits immune cells, thereby normalizing the inflammatory response. Aggregation of FcεRI triggers AD. After induction of AD, mast cells are activated by crosslinking of adjacent IgE molecules [39]. Notably, IgE is responsible for both acute and chronic symptoms in atopy-like skin diseases, and repeated injection of TMA increases serum IgE levels in the mice [40]. We found that total serum IgE levels in mice were significantly increased by repeated TMA treatment. BV treatment significantly reduced total serum IgE levels in TMA-treated mice.

Many studies suggest that an imbalance between Th1 and Th2 responses is closely associated with many immune disorders, including atopic dermatitis [41]. In typical allergic diseases, there are increases in the levels of Th2 cytokines and decreases in the levels of Th1 cytokines [42]. Many studies also support the assertion that restoration of the balance between Th1 and Th2 cell numbers, which can be achieved by either stimulation of Th1 cell development or inhibition of Th2 cell development, has remedial value in AD treatment [43]. However, interestingly, in our mouse model of atopy-like skin dermatitis, in which the expression levels of both Th1 and Th2 cytokines simultaneously increased, the alleviation of atopic skin symptoms by BV treatment was attributed to downregulation of both Th1 and Th2 cytokines. Th2-dominated cytokine profile plays a central role in the initiation of AD symptoms in the early stage, and in the chronic stage of human atopic dermatitis, cytokine profile shift from Th2 to Th1 is quite critical for aggravation of AD leading to severe dermal inflammation. Although our atopic model of TMA-induced hypersensitivity for 2 weeks seems to show such a mixed profile of both Th1 and Th2 dominance and thus be close to human chronic dermatitis of atopy, we do not have a confidence because we did not analyze time-course profiles of Th1- and Th-2 expression.

Local injection of BV exhibited simultaneous anti-inflammatory and anti-allergy effects for the treatment of atopic skin symptoms. This phenomenon might be caused by the double-sided character of BV, as mentioned earlier, although we did not clearly elucidate its mechanism in this study.

Unlike usual immunotherapies for immune diseases, venom therapy not only induces a rapid shift in cytokine expression from Th2 (IL-4) to Th1 (IFN-γ) cytokines, but also leads to increased production of immunosuppressive cytokine IL-10 during the initial phase of the treatment [44]. Furthermore, it was reported that although low-dose BV treatment initially upregulated Th1 cytokine expression in experimental immunotherapy, aggravating inflammation, pain, and scratching behavior, it eventually relieved those symptoms [45].

In the present study, BV stimulation at a non-acupuncture point also exhibited a significant suppressive effect on TMA-induced edema formation, T-cell activation and the levels of inflammatory cytokine mRNAs and proteins. It means that BV itself showed certain levels of anti-inflammatory and anti-allergic activities, regardless of the use of specific acupuncture point.

Although we addressed that BVA significantly inhibited the TMA-induced expression of Th2- and Th1- cytokines in the present study, the detailed mechanisms of how BVA modulated the expression levels of Th1- and Th2-cytokines together should be elucidated in the future.

## Conclusion

In the atopy-like dermatitis mouse model, the expression of Th2 cytokines such as IL-4, IL-5, and IL-10 in the lymph nodes of TMA-treated mice was found to be elevated, and BVA largely abrogated TMA-induced production of Th2 cytokines. Levels of Th1 cytokines (IL-2, IL-12, IFN-γ, and TNF-α) were also increased by TMA treatment, and the increases were suppressed by BVA treatment. This means that BVA significantly suppressed the expression of both Th1 and Th2 cytokines, indicating comprehensive modulation of the Th1-Th2 balance. Although the precise mechanism by which BV inhibits Th2 cytokines and Th1 cytokines in our atopic mouse model was not explicitly elucidated in this study, the remedial value of BV therapy might be attributed to not only its widely known anti-inflammatory activity, but also its strong anti-allergic activity, which we report here for the first time.

## Additional file

Additional file 1: Figure S1. Time-courses of surface temperatures at the skin sites that BV was injected in subcutaneously, intradermally or intramuscularly. BV (0.3 mg /kg body weight) was injected into the mid-back after hair removal, and the temperature at the injection site in the skin was measured using an infrared thermometer (HuBDIC Thermofinder (FS-300), Beauty Korea World Co., Ltd., Seoul, Korea). BV injections were performed in at least three mice, and temperature measurement was repeated three times per injection per time point. (DOCX 42 kb)

## Abbreviations

AD: atopic dermatitis
BV: bee venom
BVA: bee venom acupuncture
CD: cluster of differentiation
cDNA: complementary DNA
DAB: diaminobenzidine
ELISA: enzyme-linked immunosorbent assay
GAPDH: glyceraldehyde 3-phosphate dehydrogenase
GM-CSF: granulocyte-macrophage colony-stimulating factor
HRP: avidin-horseradish peroxidase
IFN: interferon
IgE: immunoglobulin E
IL: interleukin
Th1: T helper cell type 1
Th2: T helper cell type 2
TMA: trimellitic anhydride
TMB: tetramethylbenzidine
TNF: tumor necrosis factor
