# Supplementary material for: Bee venom acupuncture alleviates trimellitic anhydride-induced atopic dermatitis-like skin lesions in mice
Source: BMC Complement Altern Med. 2016 Jan 29;16:38. doi: 10.1186/s12906-016-1019-y (PMC4731956; doi:10.1186/s12906-016-1019-y)
Supplement: Additional file 1: Figure S1. — Time-courses of surface temperatures at the skin sites that BV was injected in subcutaneously, intradermally or intramuscularly. BV (0.3 mg /kg body weight) was injected into the mid-back after hair removal, and the temperature at the injection site in the skin was measured using an infrared thermometer (HuBDIC Thermofinder (FS-300), Beauty Korea World Co., Ltd., Seoul, Korea). BV injections were performed in at least three mice, and temperature measurement was repeated three times per injection per time point. (DOCX 42 kb) [file 12906_2016_1019_MOESM1_ESM.docx]

**Additional file 1**


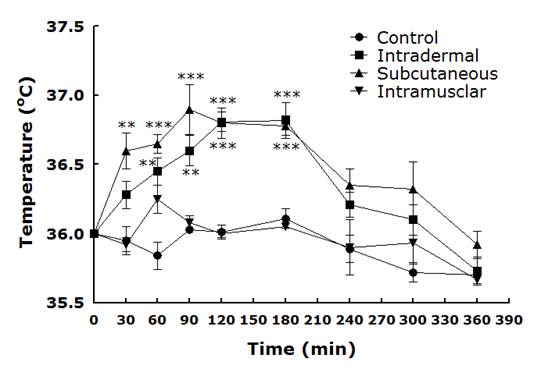


**Figure S1.** Time-courses of surface temperatures at the skin sites that BV was injected in subcutaneously, intradermally or intramuscularly. BV (0.3 mg /kg body weight) was injected into the mid-back after hair removal, and the temperature at the injection site in the skin was measured using an infrared thermometer (HuBDIC Thermofinder (FS-300), Beauty Korea World Co., Ltd., Seoul, Korea). BV injections were performed in at least three mice, and temperature measurement was repeated three times per injection per time point.

**Optimization of the BVA injection method**

To determine the best BV injection method, skin temperature at the injection site on the back were measured for subcutaneous, intradermal, and intramuscular injections of BV. One possible way to determine the optimum injection method or daily dose is the measurement of skin temperature at BV-injected sites, because BV treatment is considered to be one of immunotherapy, and thus may cause instant inflammation or an allergic response. In the present study, we measured the temperatures of injected skin sites after intradermal, subcutaneous, and intramuscular BV injections. As shown in Fig. 2, the temperature began to increase immediately after intradermal or subcutaneous injection of BV, eventually reaching a peak of 36.8°C at 2 h. In contrast, intramuscular BV injection did not significantly increase skin temperature. The skin temperature gradually decreased 3 h after injection, and returned to a normal level at 6 h with 0.3 mg BV/kg body weight, irrespective of injection method. We thus used subcutaneous BVA injection inducing a rapid increase of skin temperature around the injection point in the present study. Body temperature was not changed significantly at this BV dose (data not shown).
